# BAMBI Regulates Angiogenesis and Endothelial Homeostasis through Modulation of Alternative TGFβ Signaling

**DOI:** 10.1371/journal.pone.0039406

**Published:** 2012-06-25

**Authors:** Nicolas Guillot, Dmitrij Kollins, Victoria Gilbert, Sandhya Xavier, Jun Chen, Madeleine Gentle, Anand Reddy, Erwin Bottinger, Rulang Jiang, Maria Pia Rastaldi, Alessandro Corbelli, Detlef Schlondorff

**Affiliations:** 1 Department of Medicine, Mount Sinai School of Medicine, New York, New York, United States of America; 2 Department of Medicine, New York Medical College, Valhalla, New York, United States of America; 3 Cincinnati Children’s Hospital Medical Center, Cincinnati, Ohio, United States of America; 4 Renal Research Laboratory, Ospedale Maggior Policlinico & Fondazione D’Amico per la Ricerca sulle Malattie Renali, Milan, Italy; Institut National de la Santé et de la Recherche Médicale, France

## Abstract

**Background:**

BAMBI is a type I TGFβ receptor antagonist, whose in vivo function remains unclear, as BAMBI**^−^** ^/**−**^ mice lack an obvious phenotype.

**Methodology/Principal Findings:**

Identifying BAMBI’s functions requires identification of cell-specific expression of BAMBI. By immunohistology we found BAMBI expression restricted to endothelial cells and by electron microscopy BAMBI**^−^** ^/**−**^ mice showed prominent and swollen endothelial cells in myocardial and glomerular capillaries. In endothelial cells over-expression of BAMBI reduced, whereas knock-down enhanced capillary growth and migration in response to TGFβ. In vivo angiogenesis was enhanced in matrigel implants and in glomerular hypertrophy after unilateral nephrectomy in BAMBI**^−^** ^/**−**^ compared to BAMBI^+/+^ mice consistent with an endothelial phenotype for BAMBI**^−^** ^/**−**^ mice. BAMBI’s mechanism of action in endothelial cells was examined by canonical and alternative TGFβ signaling in HUVEC with over-expression or knock-down of BAMBI. BAMBI knockdown enhanced basal and TGFβ stimulated SMAD1/5 and ERK1/2 phosphorylation, while over-expression prevented both.

**Conclusions/Significance:**

Thus we provide a first description of a vascular phenotype for BAMBI**^−^** ^/**−**^ mice, and provide in vitro and in vivo evidence that BAMBI contributes to endothelial and vascular homeostasis. Further, we demonstrate that in endothelial cells BAMBI interferes with alternative TGFβ signaling, most likely through the ALK 1 receptor, which may explain the phenotype observed in BAMBI**^−^** ^/**−**^ mice. This newly described role for BAMBI in regulating endothelial function has potential implications for understanding and treating vascular disease and tumor neo-angiogenesis.

## Introduction

Transforming growth factor β (TGFβ) is involved in the regulation of many developmental and physiological processes, as well as in the pathophysiology of many diseases. The multiple and often opposing actions of TGFβ are due to the cell-type specific expression of the diverse members of the TGFβ family, their multiple receptors and signaling pathways, which can be further influenced by cell-restricted modulator proteins [1]–[3]. BAMBI (BMP and Activin receptor Membrane Bound Inhibitor) was described as such a modulator, with a putative function as a dominant negative, non-signaling, competitive pseudo-receptor for members of the TGFβ type 1 receptor (TβR1) family [4]–[6]. As BAMBI is co-expressed with members of the TGFβ family during development and in cancer, it was proposed, that BAMBI may play a role in development [7], [8] and in tumor growth and metastasis [9]–[11]. However the genetic elimination of BAMBI resulted in normal development, litter size, growth and survival of the mice [12], and thus the physiological function of BAMBI remains unclear. The absence of an obvious phenotype in BAMBI **^−^** ^/**−**^ mice is surprising as germ-line deletion of members of the BMP and TGFβ systems result in major abnormalities, many of them involving the vascular system [13]–[15].

We argued that the interaction of BAMBI with the TGF family would require co-expression of the respective receptors and BAMBI in the same cell type [1], [16], so genetic elimination of BAMBI would result in enhanced TGFβ activity restricted to that cell type. However the basic information on cell type-specific expression of BAMBI in mammalian organs is lacking, as only whole organ or tumor mRNA levels have been reported [9], [17]. Thus a cell-restricted gain of function phenotype in BAMBI **^−^** ^/**−**^ mice could escape detection. TGFβ plays an important role in angiogenesis and signals through alternative pathways in endothelial cells [13], [18]–[24]. Furthermore we recently noted, that in kidneys BAMBI is expressed predominantly in endothelial cells [25]. Therefore we hypothesized, that BAMBI may play a role in modulating endothelial biology.

We now report that BAMBI expression is restricted to vascular endothelial cells in all major organs examined. Results of *in vitro* and *in vivo* angiogenesis assays show that BAMBI decreases angiogenesis, whereas BAMBI elimination enhances angiogenesis under all experimental conditions. Furthermore BAMBI**^−^** ^/**−**^ mice have an endothelial phenotype as evidenced by electron microscopy of capillaries in heart and kidney tissue, and by larger renal glomerular capillary convolutions, which show enhanced neo-angiogenesis during compensatory renal hypertrophy in BAMBI**^−^** ^/**−**^ as compared to BAMBI^+/+^ mice. In HUVEC the effects of BAMBI are mediated predominantly through interactions with alternative TGFβ signaling through SMAD1/5 and ERK 1/2 phosphorylation. Taken together we identify for the first time a vascular endothelial phenotype in BAMBI**^−^** ^/**−**^ mice, and provide evidence for a physiological role for BAMBI in endothelial biology and vascular homeostasis, observations that may be of considerable interest for the modification of the vascular actions of TGFβ by BAMBI, including neo-angiogenesis during tissue injury and during tumor growth.

## Methods

A detailed Methods section can be found as an online supplement. (Methods S1).

### Ethics Statement

All animal studies were carried out with compliance with the Mount Sinai School of Medicine Institutional Animal Care and Use Committee approved protocols (protocol number LA08-00399).

### Mice

The BAMBI**^−^** ^/**−**^ mice were generated as reported [12].

### Cell Culture and Transfection

Primary human endothelial cells, HUVEC, (Lonza, Walkersville, MD) were transfected with BAMBI siRNA (Silencer selected pre-designed siRNA, Ambion, Austin, TX) using Lipofectamine RNAiMAX (Invitrogen, Carlsbad, CA), according to the manufacturers’ protocol. The endothelial cell line EA.hy926 (ATCC, Rockville, MD) was transfected with BAMBI or control lentiviral constructs as previously described [25].

### *In Vitro* Angiogenesis Assay

Endothelial cells were seeded on cytokine depleted Matrigel (BD Bioscience) and cultured with/without addition of TGFβ-1 (1 ng/mL final concentration) or vehicle at 37°C. Cells were photographed using a Nikon TE2000-U microscope equipped with a CCD camera (Diagnostic Instruments) [26].

### *In vivo* Angiogenesis

Angiogenesis was evaluated using the modification of the matrigel implant assay as described by Guedez et al. with the assay kit DIVAA TM (Trevigen, USA) [27].

### Transmission Electron Microscopy (EM)

EM was performed as previously reported [28]. The number of capillaries was evaluated in heart specimens for 10 images per organ, and expressed as number of capillaries/field. Endothelial cell number and thickness and regularity of capillary basement membranes were assessed for 20 randomly selected capillaries per specimen. In kidneys, measurements were performed on 6 randomly selected capillary loops in 5 randomly selected glomeruli per specimen.

### Unilateral Nephrectomy

The right kidney of BAMBI^+/+^ and BAMBI**^−^** ^/**−**^ male 6 weeks old mice was surgically removed as previously described [29]. After two weeks, mice were sacrificed and the remaining kidney removed for staining. Urine and blood samples were collected on the day of operation and on the day of sacrifice. Blood urea nitrogen (BUN) was measured by the commercial DIUR-500-QuantiChromUrea Assay (Gentaur, USA). Proteinuria was determined as urine albumin/creatinine ratio using ELISA (Exocell, USA).

### Statistical Analysis

Data are presented as mean ± SEM. For all assays, statistical analysis was performed using a Mann-Whitney test. For the matrigel implant and the unilateral nephrectomy experiments, where the same animal was sampled at different times, the Wilcoxon test was used. We deemed a P<0.05 as significant. We used Statview software for all analyses.

## Results

### BAMBI mRNA Levels Parallel Organ Vascularity and BAMBI Localizes to Vascular Endothelium

**Figure 1:**
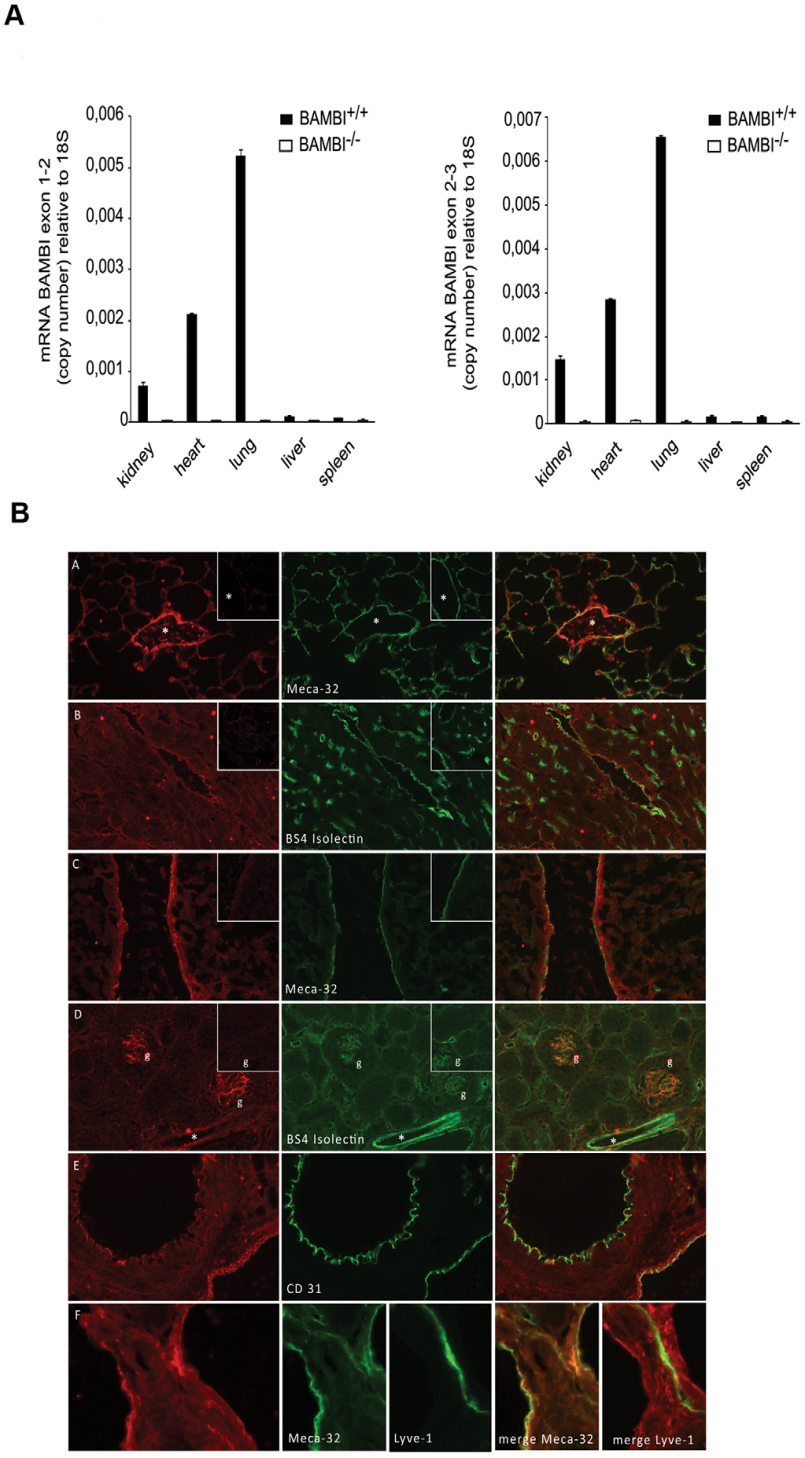
Expression of BAMBI in different mouse organs. **A.** Levels of mRNA (relative copy number) were determined in different organs from BAMBI^+/+^ and BAMBI**^−^** ^/**−**^ mice. Two different primer pairs were used, spanning either exons 2–3 (top panel) or exons 1–2 (bottom panel) in order to exclude transcripts from a potential second start site in the BAMBI gene. In the tissues from the BAMBI**^−^** ^/**−**^ mice, and using either primer pair, the levels of mRNA for BAMBI were indistinguishable from the RT minus or blank controls. Results are means ± SEM from triplicate determinations performed in two sets of mice. **B.** Immunofluorescence staining for BAMBI performed on different organs from BAMBI^+/+^ and BAMBI**^−^** ^/**−**^ mice. Organs are by rows: A: lung; B: heart; C: liver; D: kidney (g indicates glomeruli); E: femoral artery and vein; F: veins with accompagnying lymphatic vessel (LYVE-1 staining). Magnification 400x for A, B, C and F. 200x for D and E. BAMBI staining in red (first column A–F BAMBI^+/+^ mouse tissues), second column endothelial markers in green (MECA32, BS 4 Isolectin ) and the merge in the third column in yellow. BAMBI co-localizes with endothelial markers in all tissues, but is negative in tissues from BAMBI**^−^** ^/**−**^ mice (Inserts first column row A–D). Lymphatic endothelium stains with Lyve-1 antibody, but not with BAMBI antibody or MECA 32 (bottom row).

BAMBI mRNA levels using two different primer pairs were high in lung, heart and kidney, but low in liver and spleen in BAMBI^+/+^ mice, (Figure 1A). No signal for BAMBI mRNA was detected in BAMBI **^−^** ^/**−**^ mice. By immuno-fluorescence BAMBI localized to endothelial cells of arteries, veins, and capillaries in the kidney, heart, and lung. In liver, only the endothelium of blood vessels was positive for BAMBI (Figure 1B and Figure S4 for high resolution). Double staining with antibodies against endothelial cell-specific antigens of the different organs (lectin [30], MECA-32 [31], CD31 [32]) confirmed the endothelial localization (Figure 1B). Only background fluorescence was observed for BAMBI in BAMBI**^−^** ^/**−**^ tissues, confirming the specificity of the immuno-staining (Inserts Figure 1B), while staining for endothelial markers was unaffected. By double staining with LYVE1 [33], an antibody specific for lymphatic endothelium, lymphatic vessels were negative for BAMBI (Figure 1B and Figure S4). Thus BAMBI expression is restricted to vascular endothelium.

### BAMBI Influences Angiogenesis *in vitro*

**Figure 2:**
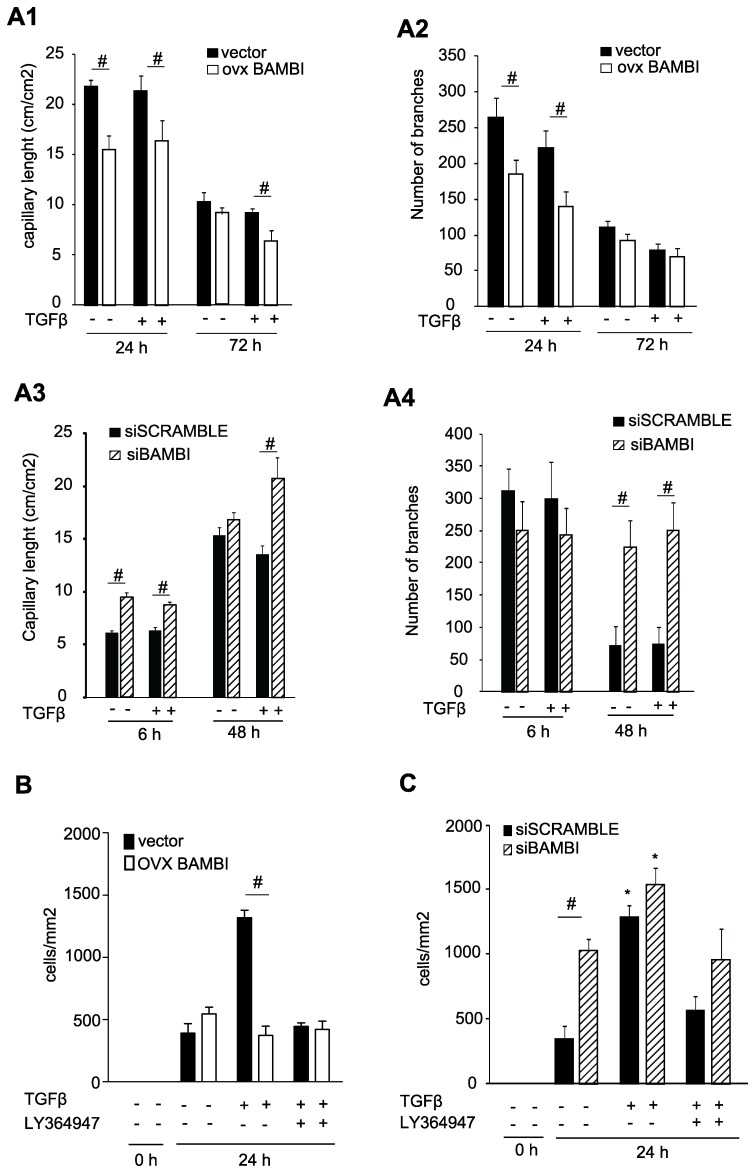
*In vitro* capillary tube formation and scratch wound closing assay with endothelial cells with knock-down or over-expression of BAMBI. **A**. Over-expression of BAMBI inhibits and knock-down of BAMBI enhances capillary tube formation in endothelial cells. **A1** and **A2**. BAMBI-over-expressing or empty vector transfected endothelial cells and **A3** and **A4**. HUVEC transfected with either scrambled RNA or siRNA for BAMBI were plated on growth factor depleted Matrigel with or without addition of TGFβ (1 ng/ml). Pictures were taken at the times indicated and capillary tube length and branching were determined. Results are means ± SEM, *n* = 3. # indicates *P*<0.05 for comparison between groups. **B and C**. Scratch wound closure is reduced by BAMBI over-expression and enhanced after knockdown of BAMBI in HUVEC. A scratch wound was created across the respective type of HUVEC and closure of the gap observed at 24 hours by determining the number of cells in the scratch-gap. Incubations were performed in the presence or absence of TGFβ and LY364947. Results are means ± SEM of 4 sets of independent experiments. # indicates *P*<0.05 for comparison between groups and * *P*<0.05 for comparison to the respective basal value.

Angiogenesis was examined *in vitro* with either a BAMBI over-expressing HUVEC cell line, or primary endothelial cells transfected with siRNA for BAMBI. As controls we used respective endothelial cells transfected with either empty vector or siScramble RNA. Over-expression of BAMBI shortened capillary formation and decreased branching independently of addition of TGFβ at 24 hours, but at 72 hours only capillary lengths were significantly decreased in the BAMBI overexpressing HUVEC treated with TGFβ (Figure 2 A1 and A2 and Figure S1 A). Knock-down of BAMBI siRNA (verified by mRNA and Western blot; Figure S2) increased capillary length with TGFβ and branching independently of TGFβ addition at 48 hours as compared to scrambled RNA (Figure 2 A3 and A4 and Figure S1 B). Taken together, BAMBI over-expression decreases, while BAMBI knock-down enhances *in vitro* angiogenesis.

### BAMBI Reduces HUVEC Migration in an *in vitro* Scratch Wound Assay

BAMBI’s effects on endothelial cells migration were evaluated by *in vitro* scratch wound assays. BAMBI over-expression did not influence the migration of HUVEC into the cell-free gap in the absence of TGFβ as compared to empty vector-transfected cells (Figure 2B). TGFβ markedly accelerated the cell migration, an effect completely inhibited by BAMBI over-expression, or by the TβRI kinase inhibitor LY364947(Figure 2B) [34]. Transfection with scrambled RNA did not influence cell migration (siSCRAMBLE *vs*. non-transfected HUVECs: 371.8±93.8 and 351.51±87.9 cells/mm^2^, respectively, *n* = 3). Knock-down of BAMBI by siRNA significantly enhanced migration in the absence of TGFβ (Figure 2C).TGFβ enhanced cell migration both in the scrambled RNA and the siBAMBI transfected HUVEC, These effects of TGFβ were inhibited by LY364947 in both scrambled and siBAMBI transfected cells (Figure 2 C). Taken together BAMBI over-expression abolishes the effect of TGFβ on closing the scratch gap, whereas BAMBI knock-down enhances scratch-wound closure.

### In vivo Angiogenesis is Enhanced in BAMB**^−^** ^/**−**^ Mice

**Figure 3:**
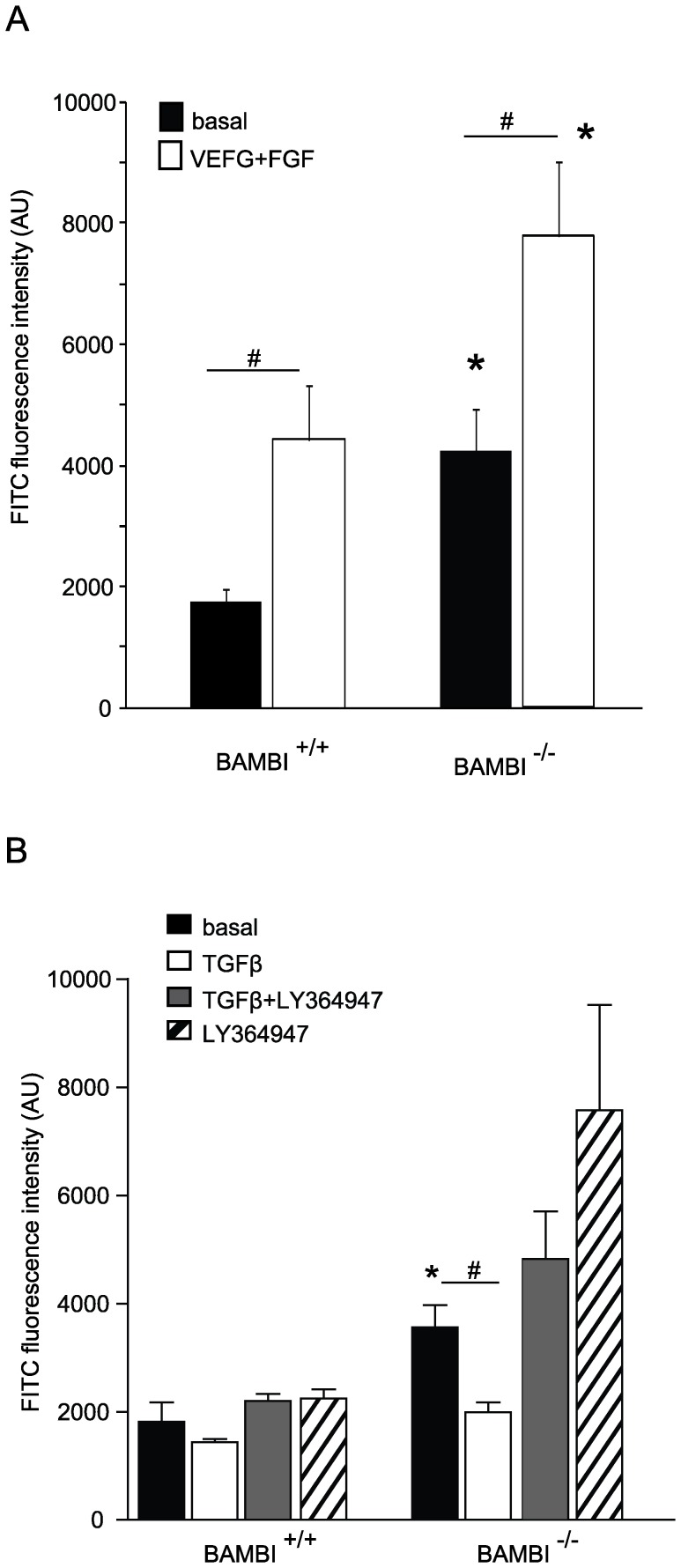
*In vivo* angiogenesis by modified-matrigel implantation assay is enhanced in BAMBI^−/−^ mice in comparison to their BAMBI^+/+^ littermates. **A.** *In vivo* angiogenesis was evaluated under basal conditions and in the presence of VEGF and FGF in the implants in BAMBI^+/+^ and BAMBI**^−^** ^/**−**^ mice. In-growth of endothelial cells was evaluated after 12 days by isolectin B4 fluorescence determination. Results are means ± SEM from 4 animals per group. # *P*<0.05 *vs*. respective basal values; * *P*<0.05 BAMBI**^−^** ^/**−**^ *vs*. BAMBI^+/+^. **B.** Comparable experiments except that TGFβ ± LY364947 or LY364947 alone were added to the matrigel implants. Results are means ± SEM from 5 mice per group. * *P*<0.05 comparing respective basal *vs.* growth factor stimulated values and # *P*<0.05 for group comparison of BAMBI^+/+^ and BAMBI**^−^** ^/**−**^ mice in the same condition.

To test the role of BAMBI on angiogenesis *in vivo*, we used implantation of silicone tubes filled with matrigel and with or without growth factors (VEGF and FGF), TGFβ or LY364947. Angiogenesis was significantly enhanced in the BAMBI**^−^** ^/**−**^ mice under basal conditions as well as with addition of VEGF and FGF (Figure 3A). Addition of TGFβ to the implants had a slight, but not significant inhibitory effect in BAMBI^+/+^ mice, but markedly inhibited the enhanced angiogenesis in BAMBI**^−^** ^/**−**^ mice (Figure 3, B). In BAMBI**^−^** ^/**−**^ mice LY364947 prevented the inhibitory effect of TGFβ (Figure 3, B). The enhancing effect of LY36494 in the BAMBI**^−^** ^/**−**^ mice, may be due to blockage of endogenous TGFβ generated in response to tube implantation. Taken together the *in vivo* and *in vitro* data show that BAMBI acts as a modulator of angiogenesis, which can be explained to a large extent by influencing endothelial effects of TGFβ.

### BAMBI **^−^** ^/**−**^ Mice Show an Endothelial Phenotype

**Figure 4:**
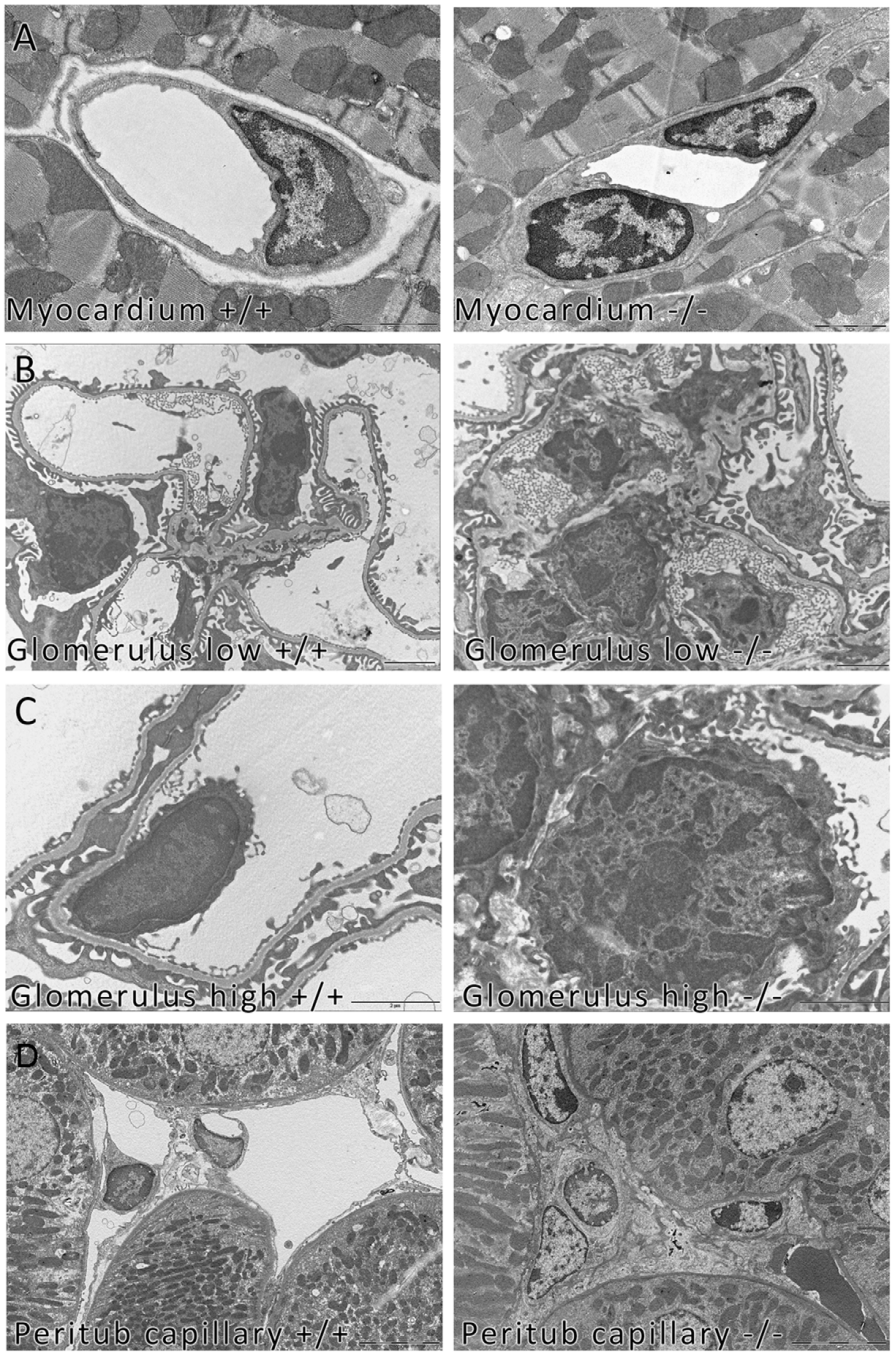
Electron microscopy pictures show an activated endothelial phenotype in tissues from BAMBI^−/−^ as compared to BAMBI^+/+^ mice. **A**. Myocardium of the BAMBI**^−^** ^/**−**^ mice is notable for the number of endothelial cells per capillary cross-section and the prominent nuclei. In BAMBI^+/+^ mice myocardial capillaries show one endothelial cell per cross-section, while those from BAMBI**^−^** ^/**−**^ mice show two nuclei per cross-section in 70% of the capillaries (scale bar 2 µm). **B** and **C**. glomeruli from BAMBI**^−^** ^/**−**^ mice show a prominent number of endothelial cells, which also appear swollen as compared to those from BAMBI^+/+^ mice. The glomerular capillary lumen is almost obliterated by the endothelial cells in the BAMBI**^−^** ^/**−**^ mice *vs.* BAMBI^+/+^ (low magnification).The activated endothelial phenotype in the BAMBI**^−^** ^/**−**^ glomeruli is also apparent at higher magnification (bottom row). **D.** Peritubular capillaries of BAMBI**^−^** ^/**−**^ mice also show a prominent and swollen endothelial cells impinging on the capillary lumen as compared to BAMBI ^+/+^ mice.

Because of their capillary density we examined kidney glomeruli and myocardium by electron microscopy. The number of myocardial capillaries did not differ between BAMBI**^−^** ^/**−**^ and BAMBI^+/+^ mice (BAMBI^+/+^38.3; BAMBI**^−^** ^/**−**^37.8 capillaries/field). However, the number of endothelial cells per capillary cross section was increased in BAMBI**^−^** ^/**−**^ mice from only one cell in 100% of capillary cross sections of BAMBI^+/+^ myocardium to two cells in 70% of BAMBI **^−^** ^/**−**^ hearts (Figure 4A). In renal glomerular capillaries of BAMBI**^−^** ^/**−**^ mice, endothelial cells were prominent as compared to BAMBI^+/+^ mice (Figure 4B). The endothelial cell bodies appeared enlarged with “activated” nuclei, which markedly impinged on the capillary lumen (Figure 4C). No changes were noted in the endothelial fenestrations, the structure, regularity or thickness of capillary basement membranes (basement membrane width: BAMBI^+/+^213.19±39.45 nm; BAMBI**^−^** ^/**−**^207.65±57.65 nm). Epithelial foot-processes were distinct but occasionally appeared broadened. The prominence of endothelial cells was also seen in the renal peritubular capillaries of the BAMBI**^−^** ^/**−**^ mice (Figure 4D).

### Glomeruli from BAMBI**^−^** ^/**−**^ Mice are Larger with Increased Endothelial Capillary Areas, Changes Further Enhanced by Compensatory Hypertrophy

**Figure 5:**
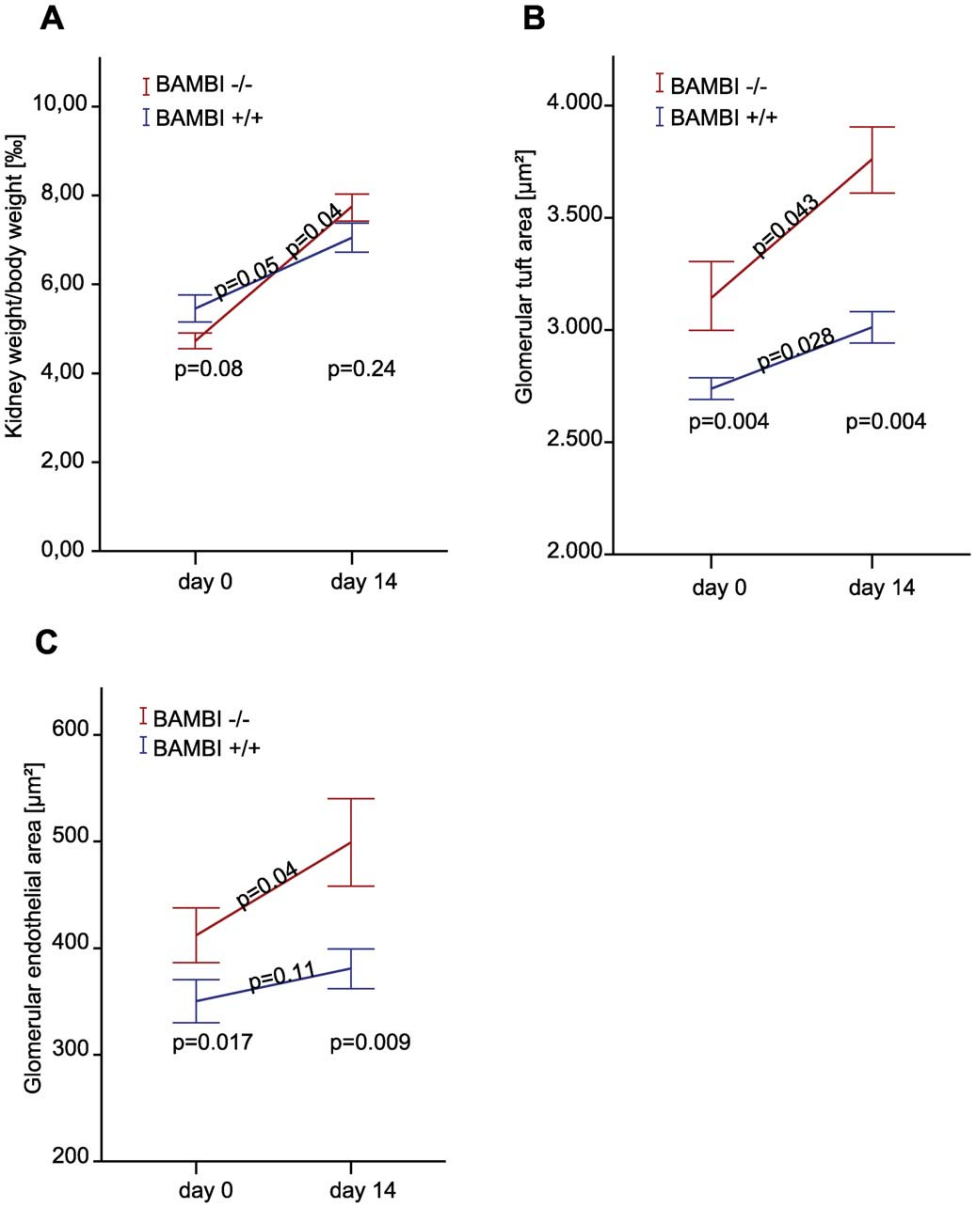
Kidney weights per body weight and glomerular and endothelial areas in the unilateral nephrectomy specimens (day 0) and in the remaining kidneys after 14 days of compensatory hypertrophy (day 14). **A.** Kidney weights per body weight are comparable between BAMBI^+/+^ and BAMBI**^−^** ^/**−**^ mice and increase to a comparable degree during the 14 days of compensatory hypertrophy **B** and **C**. Glomerular areas **B.** and endothelial (isolectin B4 positive) areas **C**. are larger in BAMBI**^−^** ^/**−**^ than in BAMBI^+/+^ mice in the unilateral nephrectomy specimens and the differences become even bigger after 14 days of compensatory renal hypertrophy. Data are mean ± SEM, *n* = 5 mice per group.

Because of the glomerular changes we examined the number, size and endothelial areas of glomeruli from adult BAMBI^+/+^ and BAMBI**^−^** ^/**−**^ mice. Total kidney weight per body weight were comparable between BAMBI^+/+^ and BAMBI**^−^** ^/**−**^ mice (Figure 5). The number of glomeruli observed by light microscopy were not different between BAMBI^+/+^ (188±9 per 4.2 mm^2^ of kidney section, *n* = 4 mice) and BAMBI**^−^** ^/**−**^ mice (204±38, *n* = 4). However, by computer-assisted morphometrical analysis of overall glomerular area (determined as area within Bowman’s space on hematoxylin and eosin stained sections) was significantly larger (*P*<0.05) in BAMBI**^−^** ^/**−**^ mice (3156±173 µm^2^ *n* = 86 glomeruli analyzed from 4 mice) as compared to age and sex matched BAMBI^+/+^ mice (2749±108 µm^2^ *n* = 87 glomeruli from 4 mice).

Total glomerular tuft area and glomerular endothelial area by iso-lectin B4 staining were determined in parallel before and after unilateral nephrectomy in BAMBI^+/+^ and BAMBI**^−^** ^/**−**^ mice (Figure S3), as the resulting compensatory hypertrophy involves both TGFβ and neo-angiogenesis [35]–[37]. Figure 5 shows values for the initial, unilateral nephrectomy specimens and for those after 2 weeks of compensatory hypertrophy. Kidney weight per body weight did not differ in the initial or in the samples after compensatory hypertrophy between BAMBI^+/+^ and BAMBI**^−^** ^/**−**^ mice, and the compensatory increase in kidney to body weight was comparable (Figure 5A). In contrast, both total glomerular tufts and isolectin B4 positive glomerular endothelial areas were significantly larger in the initial nephrectomy specimens of the BAMBI**^−^** ^/**−**^ mice (Figure 5B). These differences between BAMBI ^+/+^ and BAMBI**^−^** ^/**−**^ mice were further enhanced after compensatory hypertrophy (Figure 5 B and C) with coefficients of increase for glomerular capillary areas of 20±4% in BAMBI**^−^** ^/**−**^ and 9±2% in BAMBI ^+/+^ mice (*P*<0.05).

The endothelial changes in the BAMBI**^−^** ^/**−**^ mice did not significantly influence glomerular perm-selectivity as evidenced by lack of albuminuria in BAMBI**^−^** ^/**−**^ mice aged 4 to 18 weeks or at 2 weeks after unilateral nephrectomy (Table 1). Within the limits of the quantification of the glomerular filtration rate by BUN determination, these were comparable between BAMBI**^−^** ^/**−**^ mice and sex and age matched BAMBI^+/+^ mice either at baseline or two weeks after unilateral nephrectomy (Table 1). Thus overall glomerular function as indicated by BUN and urinary albumin/creatinine ratios appeared preserved in the BAMBI**^−^** ^/**−**^ mice in spite of the glomerular capillary obstruction from the swollen endothelial cells seen by EM, resembling the partial capillary occlusion during endotheliosis in *e.g.* pre-eclampsia [38].

**Table 1:** Albumin creatinine ratio, and BUN.

Albumin/creatinine ratio (µg/mL)	Day 0	Day 14
BAMBI^+/+^	52.4±3.5 (n = 5)	41.2±8 (n = 5)
BAMBI**^−^** ^/**−**^	60.4±16.9 (n = 4)	72.6±26.2 (n = 4)
**BUN (mg/dL)**	**Basal**	**Day 14**
BAMBI^+/+^	29.1±2.8 (n = 4)	30.39±3.8 (n = 4)
BAMBI**^−^** ^/**−**^	28.4±1.3 (n = 4)	29.6±2.9 (n = 4)

Urinary albumin to creatinine ratios from BAMBI^+/+^ and BAMBI**^−^** ^/**−**^ mice at baseline and two weeks after unilateral nephrectomy. BUN levels from BAMBI^+/+^ and BAMBI**^−^** ^/**−**^ mice at baseline and 2 weeks after unilateral nephrectomy. Values are means ± SEM for the number of animals indicated in brackets.

Taken together these results provide the first evidence, that BAMBI**^−^** ^/**−**^ mice exhibit an endothelial phenotype as evidenced by their appearance on EM pictures, enhanced *in vivo* angiogenesis in a modified matrigel assay and during glomerular compensatory hypertrophy.

### Changes in BAMBI’s Expression in HUVEC Predominantly Influence Alternative TGFβ Signaling with SMAD1/5 and ERK1/2 Phosphorylation

**Figure 6:**
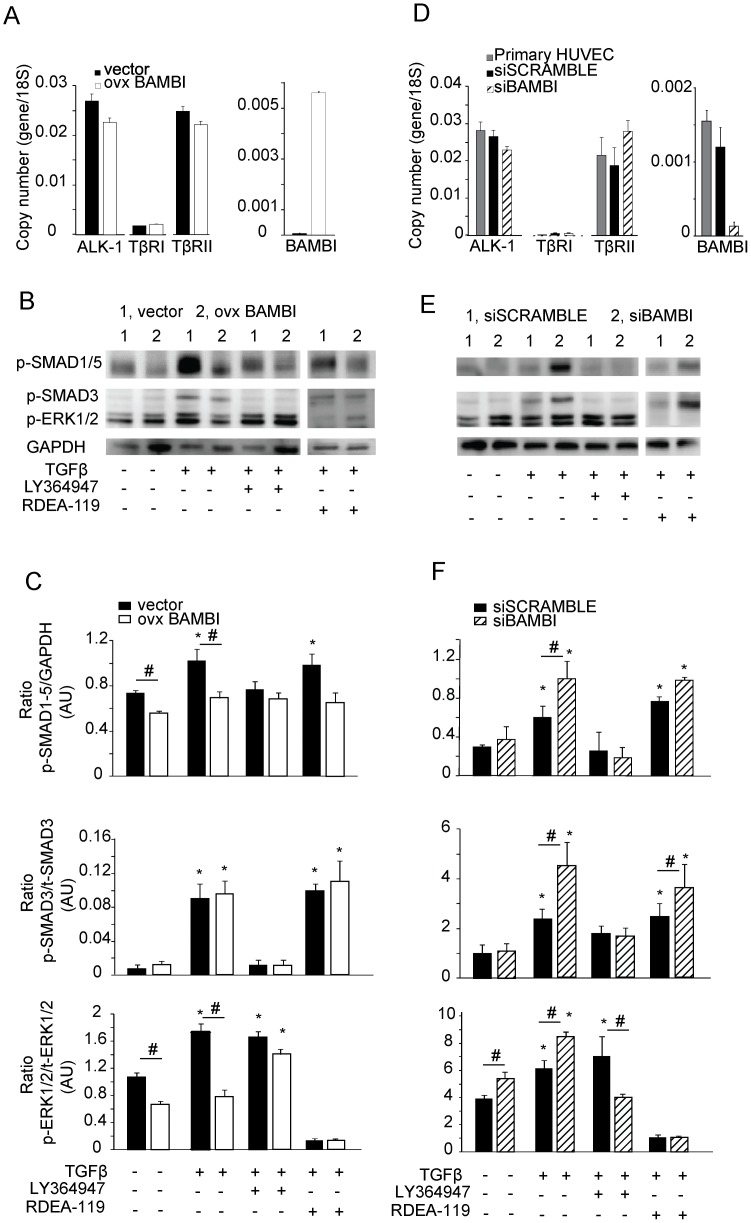
BAMBI influence alternative TGFβ signaling. **A.** and **D.** mRNA levels for BAMBI, ALK1,TβRI and TβRII in HUVEC. **A.** HUVEC cell line transfected with lentiviral system containing either the empty vector or the BAMBI over-expressing vector; **D.** primary HUVEC without or with transfection of scrambled RNA or siRNA for BAMBI knock-down). Results are means ± SEM from three experiments each and are expressed as copy number per 18S control. **B** and **E.** Analysis by Wesern blots of the phosphorylation of SMAD1/5, SMAD3 and ERK1/2 in HUVEC with either BAMBI over-expression or knock-down. **B.** BAMBI over-expression transfected or empty vector transfected HUVEC were incubated with TGFβ ± LY364947 or RDEA-119 for 15 min before extraction of the proteins and analysis by Western blot as detailed in Methods. Each blot was analyzed with antibodies specific for the phosphorylated and the total form of proteins and GAPDH, to calculate the ratio of phospho- to total protein. A representative blot is shown. The experiments with LY364947 and RDEA-119 were carried out in parallel and run on separate blots. For convenience of presentation, the lanes from the blots containing the TGFβ plus RDEA-119 samples were cut out and added to the respective blots from the parallel experiment with LY364947. **E**. Comparable experiments as in (**B**), except that HUVEC had been transfected with either scrambled siRNA or siRNA for BAMBI prior to the experimental incubations. **C** and **F.** The bar graphs show the results of the activation ratios for the different signaling molecules calculated from the densitometry readings. Each bar represents the mean ± SEM of 6 separate series of experiments carried out at basal and TGFβ stimulated conditions, in 4 series LY364947 was tested in addition, and in 3 series RDEA-119. # refers to *P*<0.05 by group comparison, and * to *P*<0.05 by comparison to the respective basal values.

BAMBI can modify effects of the TGFβ family by acting as a competitive inhibitor for type 1 TGFβ receptors [4]. However TGFβ signaling is highly cell type specific, involving both canonical SMAD2/3 as well as alternative pathways [1], [2], [39], in part through use of different TGFβ receptor combinations, which in endothelial cells includes ALK1 [2], [18]–[20], [22], [40], [41]. We therefore first examined the expression of ALK1, TβRI and TβRII and BAMBI in the different HUVEC used for signaling experiments (Figure 6A and D). All HUVECs showed robust mRNA expression of ALK1 and TβRII, but low levels for TβRI, irrespective of over-expression or knock-down of BAMBI (Figure 6A and D) consistent with reported endothelial cell-specific expression of ALK1 [2], [18]–[20], [22], [40], [41]. The canonical TGFβ pathway was analyzed by phosphorylation of SMAD3, which exceeded SMAD2 expression in HUVEC, as also noted by others [41]. SMAD1/5 phosphorylation was used as a read out for alternative TGFβ signaling [2], [19], [22]. As an additional downstream indicator of alternative TGFβ signaling, we evaluated ERK1/2 phosphorylation, which may modulate TGFβ effects on angiogenesis and vascular biology [39], [42]–[47].

Over-expression of BAMBI decreased basal and TGFβ-induced SMAD1/5 phosphorylation (Figure 6 B and C). Phosphorylation of ERK1/2 was modestly but consistently increased by TGFβ in controls (Figure 6), as also reported by Lee *et al*. [41]. Over-expression of BAMBI essentially abolished the TGFβ-induced phosphorylation of ERK1/2 (Figure 6 B and C). Both basal and TGFβ stimulated phosphorylation of ERK1/2 were completely inhibited by RDEA-119, a MAP kinase inhibitor for ERK [48]. RDEA-119 had no effects on either SMAD3 or SMAD1/5 phosphorylation (Figure 6 B and C). TGFβ also activated the canonical signaling pathway by SMAD3 phosphorylation, but this was not consistently influenced by BAMBI over-expression (Figure 6 B and C). The TβRI kinase inhibitor, LY364947, inhibited the SMAD1/5 and SMAD3 phosphorylation in both control and BAMBI over-expressing cells (Figure 6 B and C). The reasons for the inhibition of SMAD1/5 by LY364947 are unclear, but could be due to direct ALK1 kinase inhibition [34]. LY364947 did not consistently alter the response of ERK1/2 phosphorylation by TGFβ in either control or BAMBI over-expressing cells (Figure 6 B and C).

BAMBI knock-down markedly enhanced alternative TGFβ signaling with phosphorylation of SMAD1/5 and ERK1/2 (Figure 6 E and F), but SMAD3 phosphorylation also increased. Phosphorylation of SMAD3 and SMAD1/5 were inhibited by LY364947 and that of ERK 1/2 by RDEA-119 (Figure 6 E and F). LY364947 also prevented the enhanced ERK1/2 phosphorylation by TGFβ in the HUVEC with BAMBI knock-down. Thus the inhibition of SMAD1/5 phosphorylation by LY364947 may result in a downstream inhibition of ERK1/2 phosphorylation, potentially indicating a downstream effect of SMAD1/5 on ERK activation.

Taken together, BAMBI in endothelial cells interferes predominantly with the alternative TGFβ signaling pathway involving SMAD1/5 and ERK1/2. We propose that endothelial cell restricted expression of both BAMBI and ALK1 favors alternative signaling of TGFβ, which may contribute to the effects of BAMBI and TGFβ in endothelial biology that we observed both *in vitro* and *in vivo*.

## Discussion

Our studies define for the first time a vascular endothelial phenotype for BAMBI**^−^** ^/**−**^ mice. We demonstrate that in wild type mice expression of BAMBI is restricted to endothelial cells, where it acts to limit angiogenesis by interfering with alternative pathway of TGFβ signaling and thereby contributes to normal endothelial homeostasis. Consequently genetic elimination of BAMBI results in a vascular phenotype with prominent endothelial cells in myocardial and glomerular capillaries, larger glomerular capillary convolutions, and enhanced glomerular endothelial hypertrophy after unilateral nephrectomy. Furthermore, *in vivo* angiogenesis assays using matrigel implants show enhanced neo-angiogenesis in BAMBI**^−^** ^/**−**^ mice as compared to BAMBI^+/+^ mice. *In vitro* angiogenesis assays further support BAMBI’s homeostatic role in endothelial cells in that over-expression of BAMBI reduces, whereas knock-down enhances the formation of capillary networks and the closing of *in vitro* scratch wounds in HUVEC. HUVEC express ALK1 rather than TβRI, and TGFβ activates predominantly alternative signaling pathways with SMAD1/5 and ERK1/2 phosphorylation. Up or down-regulation of BAMBI in endothelial cells influences basal and TGFβ stimulated SMAD1/5 and ERK1/2 phosphorylation, which are enhanced by knock-down of BAMBI and inhibited by BAMBI over-expression. Thus we identify a physiological role for BAMBI in the modulation of endothelial biology and angiogenesis through interaction with alternative pathway activation of TGFβ involving SMAD1/5 and ERK1/2 phosphorylation.

The restricted expression of BAMBI to endothelial cells and its interference with alternative pathways of TGFβ signaling in endothelial cells are especially intriguing as TGFβ effects in endothelial cells are highly specific. This specificity is mediated through the use of different TGFβ receptors, such as ALK1, and involve predominantly non-canonical, alternative TGFβ signaling [13], [15], [20], [22], [49]. The importance of ALK1 and SMAD1/5 for endothelial/vascular function is demonstrated by the observations, that genetic elimination of either ALK1 or SMAD5 is embryonic lethal due to cardiovascular defects [13], [50] and that ablation of ALK1 decreased tumor angiogenesis in adult mice [51]. In contrast, conditional genetic elimination of TβRI in adult mice does not cause a notable vascular phenotype [13], [52]. Finally, the contribution of ALK1 signaling in endothelial-vascular modeling is supported by the observations that mutations of ALK1 have been found in patients with type II hereditary hemorrhagic teleangiectasias [38]. The predominant expression of BAMBI and ALK1 in endothelial cells may, therefore, be ideally suited to fine tune TGFβ effects on the vasculature.

Alternative TGFβ signaling can also activate neo-angiogenesis through ERK1/2 phosphorylation [42], [43], [53] and ERK1/2 activation in vascular remodeling and disease has recently attracted increased interest [43]–[45], [47], [54], [55]. We demonstrate that, in endothelial cells, endogenous BAMBI interferes with these pathways influencing *in vitro* and *in vivo* angiogenesis. BAMBI predominantly influences the ALK1 mediated activation of SMAD1/5 and also alternative pathway activation leading to ERK1/2 phosphorylation in response to TGFβ in HUVEC. Thus elimination of BAMBI in endothelial cells *in vitro* or *in vivo* tips the balance towards a pro-angiogenic TGFβ response by favoring non-canonical pathways such as phosphorylation of SMAD1/5 and of ERK1/2.

Viewing our results on the vascular endothelial effects of BAMBI in the scheme of TGFβ signaling in endothelial cells may explain our experimental observations. Over-expression or knock-down of BAMBI in HUVEC respectively inhibited or enhanced *in vitro* capillary tube formation, and migration in scratch wound assays. These effects would be expected, if increased BAMBI would inhibit and decreased BAMBI would enhance signaling through SMAD1/5 and also ERK1/2 in endothelial cells, which in fact we observed. The parallelism of the signaling experiments with the *in vitro* endothelial experiments also extends to the *in vivo* angiogenesis experiments in BAMBI^+/+^ and BAMBI**^−^** ^/**−**^ mice. Elimination of BAMBI enhanced neo-angiogenesis both under basal and growth-factor VEGF and FGF-stimulated conditions, indicating that BAMBI acts in an angio-static manner and thus loss of BAMBI enhances neo-angiogenesis by activating endothelial cells. Addition of LY364947 to the matrigel implants prevented the angio-static effect of TGFβ (Figure 3). We interpret this as consistent with reports that the *in vitro* angio-static effects of TGFβ are mediated through activation of the canonical SMAD2/3 pathway [15], [18], [19]. When the canonical pathway is inhibited by LY364947, the alternative TGFβ pathways may predominate, leading to a further enhancement in neo-angiogenesis in BAMBI**^−^** ^/**−**^ mice. The effects of LY364947 in the absence of exogenous TGFβ, may be due to the unavoidable endogenous TGFβ generation due to the wound created at the site of the implant.

These considerations are also supported by our demonstration of a vascular endothelial phenotype in BAMBI**^−^** ^/**−**^ mice. The phenotype is evident in the EM pictures of small and large blood-vessels showing activated endothelial cells with prominent nuclei. In the myocardium this translates into an increase in endothelial cells with 70% of capillary cross-sections of BAMBI**^−^** ^/**−**^ mice showing 2 endothelial cells, versus only 1 in the BAMBI^+/+^ mice. In order to quantify the vascular endothelial changes we took advantage of the high endothelial cell density in renal glomerular capillary convolutions, which express high levels of BAMBI, as previously reported by us [25]. Indeed glomerular size was larger in kidneys from BAMBI**^−^** ^/**−**^ mice than from their BAMBI^+/+^ littermates. These differences were also reflected by the larger glomerular endothelial area in the BAMBI**^−^** ^/**−**^ kidneys as compared to those in BAMBI^+/+^ kidneys, as determined by staining with the endothelial-specific iso-lectin B4 or CD31 (Figure 5). To further evaluate *in vivo* angiogenesis, we examined compensatory hypertrophy of the remaining kidney two weeks after unilateral nephrectomy, when hypertrophy is mostly completed [35], [36]. Angiogenesis with an increase in the capillary area is a major contributor to the compensatory hypertrophy in glomeruli [35], [36]. As expected glomerular size and endothelial area increased in both BAMBI^+/+^ and BAMBI**^−^** ^/**−**^ mice (Figure 5), but the change in capillary and endothelial area was significantly larger in the BAMBI**^−^** ^/**−**^ than in the BAMBI^+/+^ mice, consistent with enhanced neo-angiogenesis in the absence of BAMBI.

Why then do BAMBI**^−^** ^/**−**^ mice not show a more pronounced vascular phenotype? The reason for this may lie in the endothelial-specific expression and action of BAMBI, so that its elimination will influence endothelial cells, but will not directly affect vascular smooth muscle cells or pericytes, which play an important part in vasculogenesis [50]. Thus the changes remain restricted to prominent endothelial cells in a “gain of function” phenotype, but not in a loss of function vascular phenotype characteristic for mice with genetic elimination of TGFβ. Furthermore, endothelial homeostasis is maintained by a balance between the pro-angiogenic alternative TGFβ pathways and the anti-angiogenic canonical one. Elimination of BAMBI would shift the balance between pro- and anti-angiogenic signaling towards a pro-angiogenic one, which could account for the endothelial phenotype, that we observed in the BAMBI**^−^** ^/**−**^ mice. We conclude that changes in endothelial BAMBI levels perturb endothelial homeostasis and could thereby modify vascular remodeling in tissue injury, in a variety of diseases affecting the vascular endothelium, such as atherosclerosis, diabetes mellitus, and kidney disease, and even in the neo-angiogenesis of tumor growth.

## Supporting Information

Figure S1 **Representative pictures of in vitro capillary tube formation.** **A**, BAMBI over expressing endothelial cells and theirs control at 24 h and 72 h, treated with or without TGFβ, and **B**, endothelial cells transfected with either scrambled RNA or siRNA BAMBI at 6 h and 24 h treated with or without TGFβ
(TIF)

Figure S2 **Levels of mRNA for BAMBI in HUVEC after transfection with either scrambled siRNA or siRNA for BAMBI.** Comparable results were obtained in three independent series of experiments. Western blot for BAMBI and GAPDH from HUVEC treated as above.
(TIF)

Figure S3 **Illustration for the method used for determinations of glomerular area, capillary tuft and endothelial, isolectin B4 positive areas in slides from kidney tissue.** For the determination of capillary endothelial tuft, images of frozen sections of renal cortex were taken at 20-fold magnification, first using the appropriate fluorescence channel for the isolectin B4 marker (FITC, 516–565 nm) and second using differential interference contrast microscopy settings (DIC Nomarski). The left panel shows the image obtained with DIC-Nomarski microscopy, the central one the determination of the capillary tuft and the right picture the isolectin B4 positive area which was determined by an observer blinded to the origin of the slides using METAMORPH computer analysis.
(TIF)

Figure S4 **Immunofluorescence staining for BAMBI performed on different organs from BAMBI^+/+^ and BAMBI^−/−^ mice.**
(TIF)

Methods S1 (DOC)
