# Supplementary figures and images for: BAMBI Regulates Angiogenesis and Endothelial Homeostasis through Modulation of Alternative TGFβ Signaling
Source: PLoS One. 2012 Jun 25;7(6):e39406. doi: 10.1371/journal.pone.0039406 (PMC3382616; doi:10.1371/journal.pone.0039406)

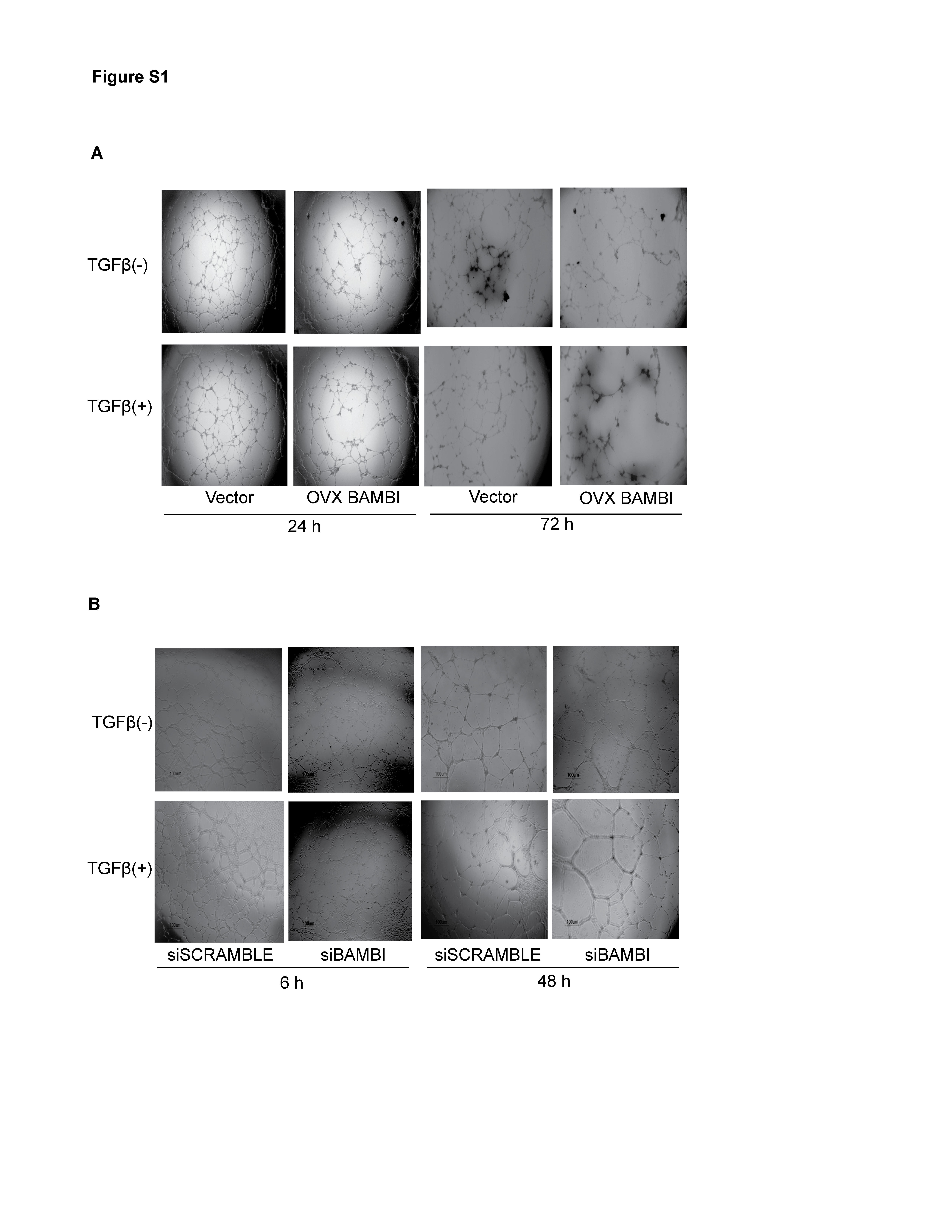

Supplement: Figure S1 — Representative pictures of in vitro capillary tube formation. A, BAMBI over expressing endothelial cells and theirs control at 24 h and 72 h, treated with or without TGFβ, and B, endothelial cells transfected with either scrambled RNA or siRNA BAMBI at 6 h and 24 h treated with or without TGFβ (TIF) [file pone.0039406.s001.tif]

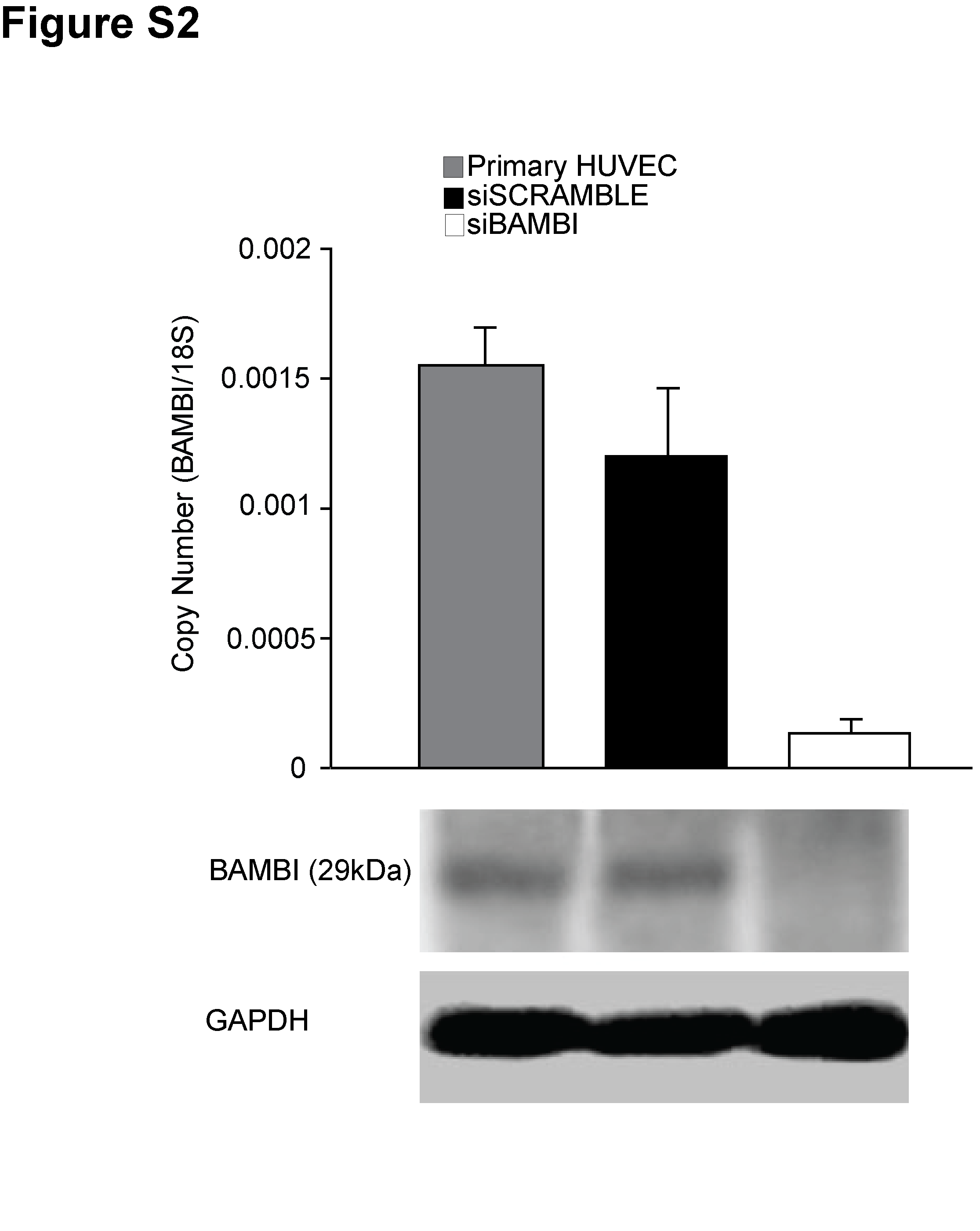

Supplement: Figure S2 — Levels of mRNA for BAMBI in HUVEC after transfection with either scrambled siRNA or siRNA for BAMBI. Comparable results were obtained in three independent series of experiments. Western blot for BAMBI and GAPDH from HUVEC treated as above. (TIF) [file pone.0039406.s002.tif]

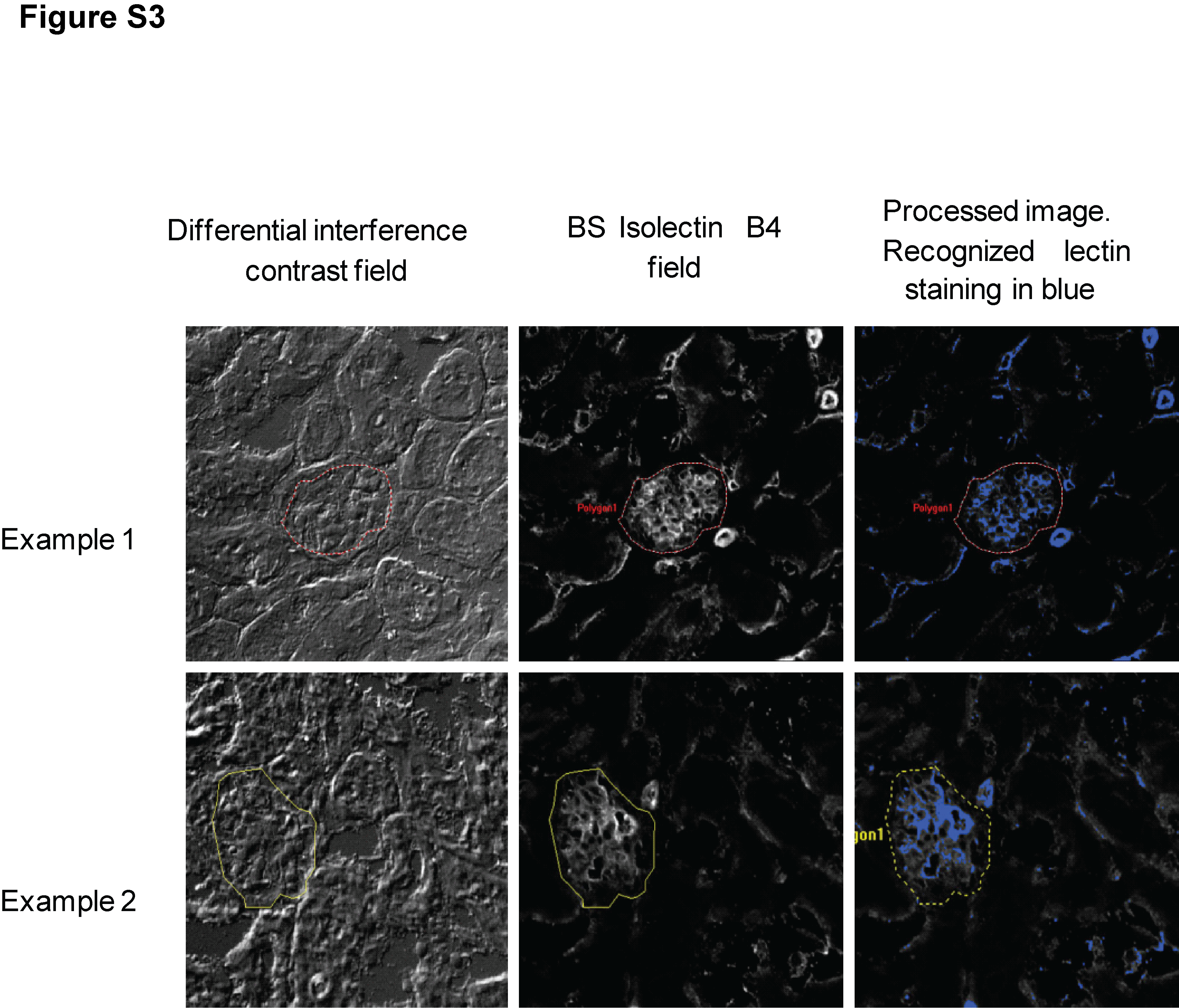

Supplement: Figure S3 — Illustration for the method used for determinations of glomerular area, capillary tuft and endothelial, isolectin B4 positive areas in slides from kidney tissue. For the determination of capillary endothelial tuft, images of frozen sections of renal cortex were taken at 20-fold magnification, first using the appropriate fluorescence channel for the isolectin B4 marker (FITC, 516–565 nm) and second using differential interference contrast microscopy settings (DIC Nomarski). The left panel shows the image obtained with DIC-Nomarski microscopy, the central one the determination of the capillary tuft and the right picture the isolectin B4 positive area which was determined by an observer blinded to the origin of the slides using METAMORPH computer analysis. (TIF) [file pone.0039406.s003.tif]

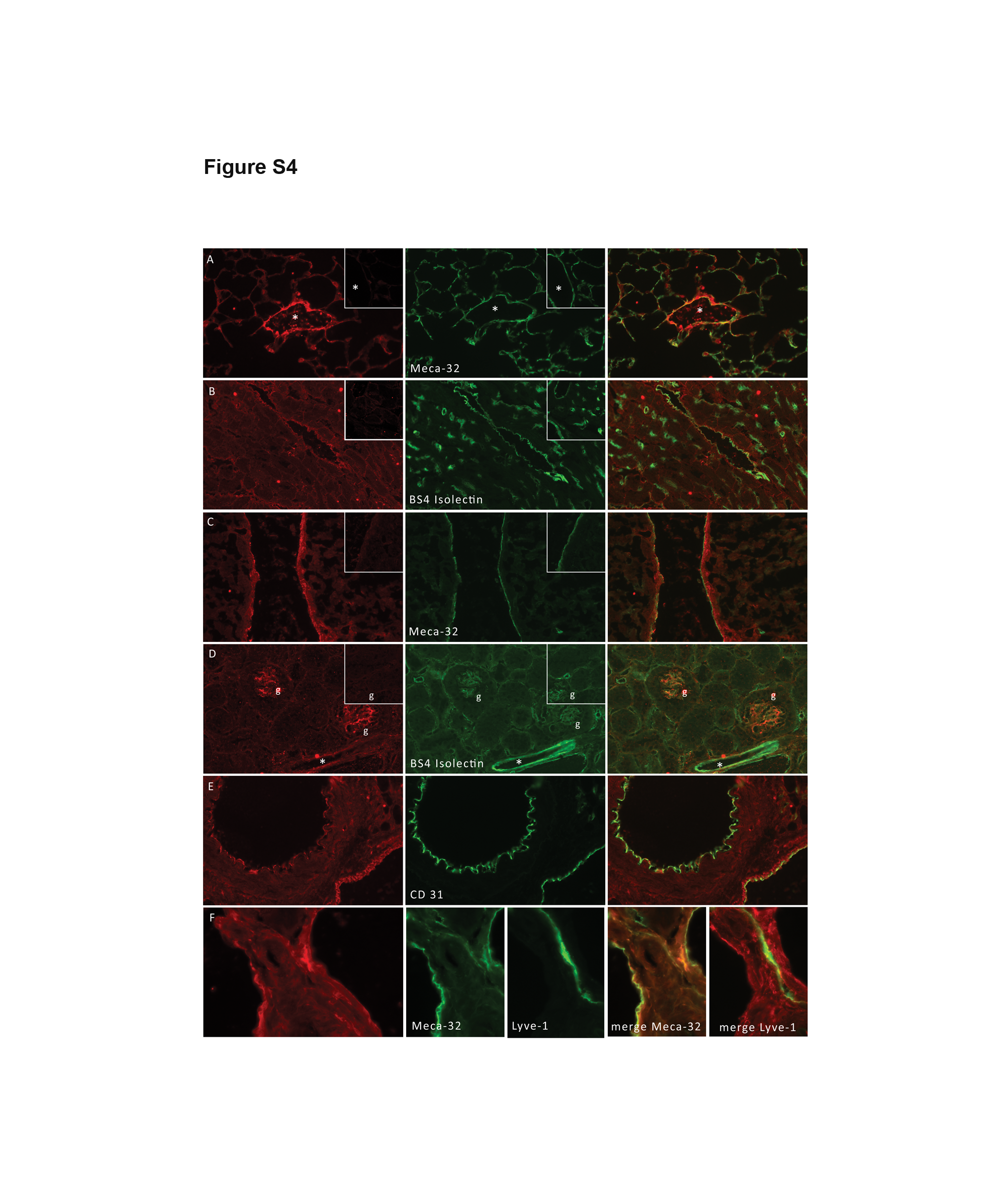

Supplement: Figure S4 — Immunofluorescence staining for BAMBI performed on different organs from BAMBI+/+ and BAMBI−/− mice. (TIF) [file pone.0039406.s004.tif]
