# Supplementary material for: BAMBI Regulates Angiogenesis and Endothelial Homeostasis through Modulation of Alternative TGFβ Signaling
Source: PLoS One. 2012 Jun 25;7(6):e39406. doi: 10.1371/journal.pone.0039406 (PMC3382616; doi:10.1371/journal.pone.0039406)
Supplement: Methods S1 — (DOC) [file pone.0039406.s005.doc]

**METHODS S1**

***Ethics Statement****:*

All animal studies were carried out with compliance with the Mount Sinai School of Medicine Institutional Animal Care and Use Committee approved protocols (protocol number LA08-00399).

***Reagents and Antibodies.*** Recombinant TGFβ-1 was from R&D systems (Minneapolis, MN). The selective inhibitor of TGFβ receptor type I, LY364947, was from Cayman Chemical (Ann Arbor, MI). The selective MEK1/2 inhibitor, RDEA-119 [1], was obtained from Active Biochemicals Co. Limited (HongKong). For immunohistology, primary antibodies used were: goat polyclonal anti-mouse BAMBI (R&D Systems, Minneapolis, MN), mouse monoclonal anti-human BAMBI (Abnova, Taiwan), Rat monoclonal anti-mouse CD31 (ab7388, Abcam, Cambrige, MA), mouse monoclonal anti-rabbit GAPDH (Santa Cruz Biotechnology, CA), sheep polyclonal anti mouse vWF (ab11713, Abcam, Cambridge, MA), rat monoclonal anti mouse MECA-32 (sc-19603, Santa Cruz, CA), Isolectin B4 (FL1201 VectorLabs, Burlingame, CA), rabbit polyclonal anti mouse LYVE-1 (ab14917, Abcam, Cambrige, MA). The secondary antibodies used in these studies were: chicken-anti-goat AlexaFluor 488 and 594 (Invitrogen), rabbit-anti-rat AlexaFluor 488 and 594 (Invitrogen), donkey-anti-sheep AlexaFluor 488 (Invitrogen), goat and donkey-anti-rabbit AlexaFluor 488 (Invitrogen). For western blot total and phospho-ERK1/2, SMAD2/3 and SMAD1/5 antibodies were from Cell Signaling (Danvers, MA), and GADPH antibody was from Santa Cruz Biothechnology (Santa Cruz, CA).

***Mice.*** The BAMBI^-/-^ mice were generated as reported [2]. BAMBI^-/-^ mice used had been backcrossed onto C57BL/6 background for 6 generations, which corresponded to a 97.3% C57BL/6 background as determined by a medium density mouse linkage SNP panel consisting of 1449 loci. For all experiments sex- and age- matched BAMBI^-/-^ and BAMBI^+/+^ mice from heterozygous intercrosses were used. . All animal studies were carried out with compliance with the Mount Sinai School of Medicine Institutional Animal Care and Use Committee approved protocols (protocol number LA08-00399).

***Quantitative Real-Time PCR analysis.*** Total RNA was isolated from all major visceral organs of BAMBI^-/-^ and BAMBI^+/+^ mice and from cultured cells and analyzed by RT-qPCR as previously reported [3]. Two different primer-pairs for BAMBI were used (forward 1 AACAGGCCCAAAACCACTCTG and reverse 1 TTGTCCTGAGGCTTCGCTCTT, forward 2 TGCACGATGTTCTCTCTCCT and reverse 2 GAAGTCAGCTCCTGCACCTT) with GAPDH, actin, and 18S as housekeeping genes to normalize the sample amount. All determinations, minus RT controls, were run in parallel and were comparable to the blanks. Fold change in gene expression was calculated by the delta-Ct method as previously described [4]. Relative copy number was determined during the qPCR assay using the Applied Biosystems Sequence Detection Software and a standard dilution curve to establish a linear relationship between Ct value and copy number in each reaction.

***Cell culture and cell transfections****.* Primary Human Umbilical Vein Endothelial Cells, HUVEC, were from Lonza (Lonza, Walkersville, MD) and cultured according to the manufacturer’s protocol with medium EBM-2 containing 2% FBS, VEGF 0.1% and FGF 0.4%. Cells were used up to five passages. These cell were transfected with 10 nM BAMBI siRNA (Silencer selected pre-disagned siRNA, from Ambion, Austin, TX ) using Lipofectamine RNAiMAX (Invitrogen, Carlsbad, CA), according to the manufacterer’s protocol. As control we used HUVEC transfected in parallel with non-targeting scrambled RNA .The effect of the siRNA or scrambled RNA transfections were monitored by mRNA determination with RT PCR and by protein analysis by Western blot for BAMBI (supplementary Figure 2). The human endothelial cell line EA.hy926 was obtained from ATCC (American Type Culture Collection, ATCC, Rockville, MD) and was maintained in Dulbecco's modified Eagle's medium (DMEM) supplemented with 10% heat-inactivated FBS, penicillin (100 U/mL) and streptomycin (50 mg/mL). This cell line was used for the lentiviral transfection system for BAMBI or empty vector expression as described previously by us [3]. Infected cells were monitored by expression of IRES-GFP from the bicistronic lentiviral vector that was under the control of an internal spleen focus-forming virus (SFFV) promoter and transfection and over-expression efficiency were monitored as previously reported [3].

***Western blot.*** Confluent endothelial cells were pre-treated overnight under serum starved conditions (0.2% FBS), followed by 15 min pretreatment with either vehicle or the TβRI kinase inhibitor LY364947 (3 μM) or the MAP kinase inhibitor RDEA-119 (10 µM) and then treated with TGFβ1 (1 ng/mL) for 15 min. Then cells were washed with ice-cold PBS and lysed with ice-cold lysis buffer (1M Tris pH7.4; 5M NaCl; 0.5M EDTA pH 7.4; 10%SDS containing Complete Mini protease inhibitor cocktail (Roche) and phosphatase inhibitor (ThermoFisher scientific, Pittsburgh, PA) Cells extracts were boiled in 2% sodium dodecyl sulfate (SDS), 1% β-mercaptoethanol, 0.008% bromophenol blue, 80mM Tris pH 6.8, 1mM EDTA. The proteins were then resolved by sodium dodecyl sulfate–polyacrylamide gel electrophoresis (SDS-PAGE) and transferred to polyvinylidene difluoride (PVDF) membranes. Membranes were incubated in blocking solution (5% albumin in Tris-buffered saline 0.1% Tween 20) for 1 h at room temperature. Membranes were washed and incubated with antibodies directed against either the total or the phosphorylated form of protein of interest. Peroxidase-labeled antibodies were used as secondary antibody. Membranes were finally visualized by enhanced chemiluminescence (Thermo Scientific) and quantified using Image Gauge Fujifilm 4.0 software.

***In Vitro* *Angiogenesis Assay.*** Endothelial cells were seeded on cytokine depleted Matrigel (BD Bioscience) and cultured with/without addition of TGFβ-1 (1ng/mLfinal concentration) or vehicle at 37°C. Cells were photographed using a Nikon TE2000-U microscope equipped with a CCD camera (Diagnostic Instruments). Photographs were analysed using Metamorph software (Universal Images, USA). For each image the number of branch points were calculated. The capillaries were traced and average integrating length of the capillary-like network was measured at different time points from at least three independent experiment using 4-5 images for each experiment [5].

***Scratch wound migration assay*.** The migration capacity of endothelial cells was analyzed by the use of the scratch wound migration assay [6]. A linear scratch was generated by drawing a pipette tip across a confluent cell monolayer grown on 24 well plates. The culture wells were then gently washed three times with PBS at 37°C and cultured at 37°C in their regular media with or without TGFβ and LY364947 as indicated. Pictures were taken at the indicated time points and the number of cells present in the wounded area was analyzed with ImageJ software.

***In vivo angiogenesis.*** Angiogenesis was evaluated using the modification of the matrigel implant assay as described by Guedez *et al.* with the assay kit DIVAA TM (Trevigen, USA) [7]. Silicone tube implants were filled with growth-factor depleted basement membrane extract with addition of either fibroblast growth factor (FGF, 1.3 μg/mL) and vascular endothelial growth factor (VEGF, 0.5 μg/mL) as positive control for angiogenesis, or PBS as negative control, TGFβ-1 (1ng/mL) or LY364947 (3μM) or TGFβ-1 plus LY364947. For each condition duplicate tubes (with a total of 4 tubes per mouse) were implanted subcutaneously into 8-week-old BAMBI^-/-^ and BAMBI^+/+^ mice. Mice were sacrificed 12 days later and neo-angiogenesis evaluated by quantification of endothelial cells that had invaded the matrix containing silicone tubes. Quantification of endothelial cells was by FITC-labeled Grifonia lectin according to the manufacturer’s protocol.

**Unilateral nephrectomy** was performed as described by Flyvbjorg *et al.* [8]. After two weeks, mice were sacrificed and the remaining kidney removed. From each kidney one part was immediately embedded in OCT medium (Tissue-Tek, USA.) and snap frozen in iso-pentane immersed in liquid nitrogen for preparation of frozen sections. Another part was fixed in 10% formalin subsequently embedded in paraffin. Horizontal cortical hematoxylin-eosin sections were observed at 10 or 20 fold magnification for morphometric analysis. All glomeruli showing both glomerular capillary tuft and Bowman’s capsule were analyzed by an observer blinded to the genotype of the mouse and using Metamorph software (MDS Analytical Technologies).For the determination of capillary endothelial tuft, images of frozen sections of renal cortex stain with FITC-isolectin B4 were taken at 20-fold magnification using a total of 10 to 15 fields per cortical area. Pictures were analyzed using Zeiss Axiovision software and differential interference contrast microscopy settings (Supplemental Figure S3). 18 to 26 glomeruli were evaluated per kidney. Samples of urine and blood were collected on the day of operation and at the day of sacrifice.BUN was determined by the commercial DIUR-500-Quantichrom Urea assay(Gentaur,USA). Proteinuria was determined as urine albumin / creatinine ratio using ELISA (Exocell, USA.).

***Immunofluorescence***

Immunohistological studies were performed on frozen, 4µm thick slices as previously reported [3]. Briefly, frozen sections were post-fixed in cold acetone washed and blocked with a solution of 2% BSA and 0.2% Fish Gelatin (GE Healthcare) in PBS. Primary staining was performed by overnight incubation at 4°C with goat-anti-BAMBI (10 μg/mL, 1:50), rat-anti-CD31 (1μg/mL, 1:100), rat-anti-MECA-32 (1:100); sheep-anti-vWF (1:100); rabbit-anti-LYVE-1 (1:100) or isolectin B4 (1:100). Slides were washed and incubated with appropriate pre-adsorbed secondary antibodies for 1 hour at room temperature. Sections were subsequently treated with DAPI. Fluorescent images were acquired using the Zeiss Axioplan2 fluorescence microscope.

***Transmission Electron Microscopy (EM).*** EM was performed as previously reported [9]. In brief, tissues were fixed in a mixture of paraformaldehyde and glutaraldehyde in phosphate buffer 0.12M, post-fixed in osmium tetroxide 1% in cacodylate buffer 0.12M, dehydrated, and embedded in Epon-Araldite resin. After staining by uranyl-acetate and lead-citrate, tissue sections were observed under a CM10 Philips microscope (FEI, Eindhoven, Netherlands). All reagents and grids for electron microscopy were from Electron Microscopy Sciences (Società Italiana Chimici, Rome, Italy). Digital electron micrographs were acquired by SIS Megawiew III camera (Olympus Italia, Segrate, Milano) from 1100X to 25000X magnification, and marker bars on electron micrographs were used as system calibration. The software analySIS (Olympus) enabled digital measurements, where appropriate. The number of capillaries was evaluated in heart specimens on 10 images per organ, and expressed as number of capillaries/field. Count of endothelial cell number and evaluation of capillary basement membranes (thickness and regularity), were performed on 20 randomly selected capillaries per specimen. In kidneys, five glomeruli per specimen were randomly selected and measurements were performed on six randomly selected capillary loops per glomerulus.

***Statistical analysis*.** Data are presented as mean ± SEM. For all assays, statistical analysis was performed using a Mann-Whitney test. For the matrigel implant and the unilateral nephrectomy experiments, where the same animal was sampled at different times, the Wilcoxon test was used. We deemed a *P*<0.05 as significant. We used Statview software for all analyses.

1. Iverson C, Larson G, Lai C, Yeh LT, Dadson C, et al. (2009) RDEA119/BAY 869766: a potent, selective, allosteric inhibitor of MEK1/2 for the treatment of cancer. Cancer Res 69: 6839-6847.

2. Chen J, Bush JO, Ovitt CE, Lan Y, Jiang R (2007) The TGF-beta pseudoreceptor gene Bambi is dispensable for mouse embryonic development and postnatal survival. Genesis 45: 482-486.

3. Xavier S, Gilbert V, Rastaldi MP, Krick S, Kollins D, et al. (2010) BAMBI is expressed in endothelial cells and is regulated by lysosomal/autolysosomal degradation. PLoS One 5: e12995.

4. Livak KJ, Schmittgen TD (2001) Analysis of relative gene expression data using real-time quantitative PCR and the 2(-Delta Delta C(T)) Method. Methods 25: 402-408.

5. Chen J, Park HC, Addabbo F, Ni J, Pelger E, et al. (2008) Kidney-derived mesenchymal stem cells contribute to vasculogenesis, angiogenesis and endothelial repair. Kidney Int 74: 879-889.

6. Rodriguez LG, Wu X, Guan JL (2005) Wound-healing assay. Methods Mol Biol 294: 23-29.

7. Guedez L, Rivera AM, Salloum R, Miller ML, Diegmueller JJ, et al. (2003) Quantitative assessment of angiogenic responses by the directed in vivo angiogenesis assay. Am J Pathol 162: 1431-1439.

8. Flyvbjerg A, Schrijvers BF, De Vriese AS, Tilton RG, Rasch R (2002) Compensatory glomerular growth after unilateral nephrectomy is VEGF dependent. Am J Physiol Endocrinol Metab 283: E362-366.

9. Giardino L, Armelloni S, Corbelli A, Mattinzoli D, Zennaro C, et al. (2009) Podocyte glutamatergic signaling contributes to the function of the glomerular filtration barrier. J Am Soc Nephrol 20: 1929-1940.
